# A randomized, double-blind comparison of OROS^® ^hydromorphone and controlled-release morphine for the control of chronic cancer pain

**DOI:** 10.1186/1472-684X-7-17

**Published:** 2008-10-31

**Authors:** Magdi Hanna, John Thipphawong, the 118 study group

**Affiliations:** 1 Pain Research Unit, King's College Hospital, King's College London, UK; 2 Analgesics and Pain Research, 62 Park Rd, Beckenham, Kent, UK; 3 Johnson and Johnson Pharmaceutical Research Division Corporation, 1010 Joaquin Road, Mountain View, California 94043, USA

## Abstract

**Background:**

Long-acting opioid formulations are advocated for maintaining pain control in chronic cancer pain. OROS^® ^hydromorphone is a sustained-release formulation of hydromorphone that requires dosing once daily to maintain therapeutic concentrations. The objective of this study was to demonstrate the clinical equivalence of immediate-release and sustained-release formulations of hydromorphone and morphine for chronic cancer pain.

**Methods:**

200 patients with cancer pain (requiring ≤ 540 mg/d of oral morphine) participated in this double-blind, parallel-group trial. Patients were randomized to receive hydromorphone or morphine (immediate-release for 2–9 days, sustained-release for 10–15 days). Efficacy was assessed with the Brief Pain Inventory (BPI), investigator and patient global evaluations, Eastern Cooperative Oncology Group performance status, and the Mini-Mental State Examination. The primary endpoint was the 'worst pain in the past 24 hours' item of the BPI, in both the immediate-release and sustained-release study phases, with treatments deemed equivalent if the 95% confidence intervals (CI) of the between-group differences at endpoint were between -1.5 and 1.5. No equivalence limits were defined for secondary endpoints.

**Results:**

Least-squares mean differences (95% CI) between groups were 0.2 (-0.4, 0.9) in the immediate-release phase and -0.8 (-1.6, -0.01) in the sustained-release phase (intent-to-treat population), indicating that the immediate-release formulations met the pre-specified equivalence criteria, but that the lower limit of the 95% CI (-1.6) was outside the boundary (-1.5) for the sustained-release formulations. BPI 'pain now PM' was significantly lower with OROS^® ^hydromorphone compared with controlled-release morphine (least-squares mean difference [95% CI], -0.77 [-1.49, -0.05]; *p *= 0.0372). Scores for other secondary efficacy variables were similar between the two sustained-release treatments. At endpoint, > 70% of investigators and patients rated both treatments as good to excellent. The safety profiles of hydromorphone and morphine were similar and typical of opioid analgesics.

**Conclusion:**

Equivalence was demonstrated for immediate-release formulations of hydromorphone and morphine, but not for the sustained-release formulations of OROS^® ^hydromorphone and controlled-release morphine. The direction of the mean difference between the treatments (-0.8) and the out-of-range lower limit of the 95% CI (-1.6) were in favor of OROS^® ^hydromorphone.

**Trial registration:**

ClinicalTrials.gov: NCT0041054

## Background

Opioid analgesics are highly effective for the treatment of pain, enabling 85% to 95% of patients to gain functional control of their lives [1,2]. Opioid therapy is typically initiated after the failure of maximum doses of non-opioid analgesics [3] and is titrated to attain the best balance between pain relief and side effects for each patient. The European Association for Palliative Care [4] and the American Pain Society [5] advocate the use of long-acting oral agents for maintaining analgesia once individual dose requirements have been established. For these reasons, long-acting opioids have become the mainstay of chronic cancer pain therapy.

OROS^® ^hydromorphone is a unique long-acting opioid formulation that utilizes Push-Pull™ active osmotic technology developed by ALZA Corporation (Mountain View, CA, USA). The Push-Pull™ system maintains consistent hydromorphone plasma concentrations throughout the 24-hour dosing interval, providing long-lasting analgesia [6-8]. Release of the drug from the system is actively controlled by the dosage form itself, and is not significantly influenced by environmental factors such as the surrounding pH or gastric motility [9,10]. There is a minimal effect of food on the rate and extent of absorption of hydromorphone from OROS^® ^hydromorphone; in one study the mean geometric ratios of fed and fasted subjects for peak plasma concentration (C_max_) and area under the concentration-time curve (AUC) were within 20%; the median time to peak plasma concentration (T_max_) was lower under fed conditions (12 versus 16 hours), but mean plasma concentration profiles generally overlapped, especially up to 6 hours after dosing [11]. The pharmacokinetics of OROS^® ^hydromorphone are also minimally affected by alcohol; one study found that plasma hydromorphone concentrations were slightly higher after alcohol (240 mL solutions of 4%, 20%, and 40% alcohol and orange juice) compared with no alcohol, but there was no clear alcohol dose-response relationship and no dose dumping of hydromorphone occurred [12].

The primary objective of the current study was to demonstrate the clinical equivalence of hydromorphone and morphine (immediate-release [IR] and sustained-release [SR] formulations) using the 'worst pain in the past 24 hours' item of the Brief Pain Inventory (BPI). Morphine was selected as the active comparator since it is the gold standard for pain control. Controlled-release (CR) morphine is available in twice-daily and once-daily formulations and is widely used to alleviate cancer pain; in this study the twice-daily formulation (dosing every 12 hours) was used.

## Methods

### Patients

This study was a multicenter, phase III, randomized, double-blind (double-dummy), active-controlled, parallel-group, equivalence trial. It was conducted at 37 centers in Belgium, Canada, France, Germany, The Netherlands, Spain, Sweden, and the United Kingdom. The study enrolled inpatients, outpatients, and day-patients ≥ 18 years of age who had moderate to severe chronic cancer pain requiring 60 to 540 mg of oral morphine (or equivalent) every 24 hours. The criteria used for patient selection are listed in Table 1. Concomitant chemotherapy or radiotherapy was permitted. All patients who entered the trial were informed of the nature of the study, and provided written informed consent for participation. The study was conducted in accordance with the principles of the Declaration of Helsinki.

**Table 1:** Criteria for patient selection

**Inclusion criteria**	**Exclusion criteria**
• Age ≥ 18 years	• Pain not considered potentially responsive to opioids
• Presence of chronic cancer pain:	• Pain present only upon movement
◦ currently receiving strong oral or transdermal opioid analgesics (60–540 mg oral morphine or equivalent every 24 hours)	• Need for other opioid analgesics (except study medication and breakthrough pain medication) after randomization
◦ appropriate candidate for strong oral or transdermal opioid analgesics (anticipated requirement, 60–540 mg oral morphine or equivalent every 24 hours)	• Current or recent (within 6 months) history of drug and/or alcohol abuse
• Pain suitable for treatment with a once-daily formulation	• Women of childbearing potential who are pregnant or lactating, seeking pregnancy, or failing to take adequate contraceptive precautions
	• Intolerance of, or hypersensitivity to, hydromorphone or other opioids
	• Presence of GI disease of sufficient severity to likely interfere with oral analgesia (e.g., dysphagia, vomiting, no bowel movement or bowel obstruction due to impaction within 5 days of study entry, severe gut narrowing that may affect analgesic absorption or transit)
	• Use of monoamine oxidase inhibitors within 2 weeks prior to study entry
	• Investigational drug use within 4 weeks of study entry
	• Presence of conditions for which risks of opioid use outweigh potential benefits (e.g., raised intracranial pressure, hypotension, hypothyroidism, asthma, reduced respiratory reserve, prostatic hypertrophy, hepatic impairment, renal impairment, elderly and debilitated, convulsive disorders, Addison's disease)

Abbreviation: GI, gastrointestinal

### Drugs and dosages

**Figure 1:**
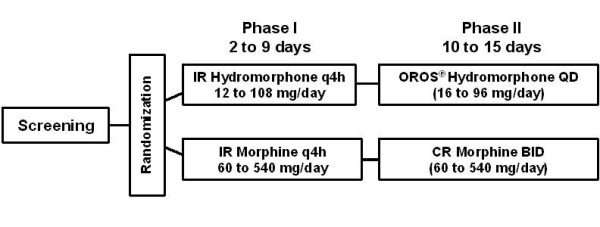
**Study design.** BID, twice daily; CR, controlled-release; IR, immediate-release; QD, once-daily; q4h, every 4 hours.

Patients were randomized 1:1, with a central computer-generated randomization list, to receive hydromorphone or morphine for up to 24 days (Figure 1). This study consisted of 2 phases: an initial IR phase and a subsequent SR phase. In the IR phase, patients received IR formulations of either hydromorphone [Dilaudid^®^, Abbott Laboratories] or morphine (morphine sulfate IR [Sevredol^®^, Napp Laboratories]) every 4 hours (6 times daily) for 2 to 9 days. Patients underwent conversion from previous therapy to 1 of 6 possible initial dose levels, based on individual patient characteristics and generally accepted morphine equivalent conversion factors. Doses were selected according to the available tablet strengths and a working conversion ratio of 1:5 (1 hydromorphone:5 morphine equivalents), in the ranges hydromorphone IR 12–108 mg/day and morphine IR 60–540 mg/day. The dose was titrated to the next higher dose level if the patient had more than 3 breakthrough pain episodes requiring breakthrough pain medication within the previous 24 hours. Daily doses were titrated up to the next higher dose level, with no dose levels skipped, at most once a day. Dose titration was continued until dose-stable pain control was achieved. Patients who experienced 2 consecutive days with no more than 3 breakthrough pain episodes requiring rescue medication per day were considered to have achieved dose-stable pain control and could begin the SR phase of the study. Patients who did not achieve dose stable pain control by day 9 were withdrawn from the study, and end-of-study evaluations were carried out.

The duration of the SR phase was 10 to 15 days. In this phase, patients continued to receive the same study drug but in an SR formulation: OROS^® ^hydromorphone once-daily or CR morphine (morphine sulfate SR [MST Continus^®^, Napp Laboratories]) twice-daily. They were started on the same dose level number on which they had achieved dose-stable pain control in the preceding IR phase, which was then adjusted as required, at most every 2 days, in single steps so that no dose level was skipped. Doses were selected according to the available tablet strengths and a working conversion ratio of approximately 1:5 (hydromorphone:morphine), in the ranges OROS^® ^hydromorphone 16–96 mg/day and CR morphine 60–520 mg/day. The SR phase was completed after a minimum of 10 days (maximum of 15 days) on OROS^® ^hydromorphone or CR morphine if pain was controlled and the dose was stable for at least the previous 2 days.

In the IR phase, the dosing regimen was the same for both drugs: patients received a dose of study medication 6 times a day, every 4 hours, at 0600, 1000, 1400, 1800, 2200, and 0200. In this phase, all treatments were over-encapsulated resulting in identical capsules. In the SR phase, dosing was done twice each day at 1000 and 2200. To maintain the blind in this phase, matching placebo tablets and capsules were used: patients in the OROS^® ^hydromorphone group took OROS^® ^hydromorphone and placebo CR morphine in the morning and placebo CR morphine in the evening; patients in the CR morphine group took CR morphine and placebo OROS^® ^hydromorphone in the morning and CR morphine in the evening.

In both study phases, patients could also receive breakthrough pain medication as needed, either hydromorphone or morphine, supplied as the IR formulation. A single dose of breakthrough pain medication contained approximately one-sixth of the patient's daily dose.

### Assessments

Baseline efficacy assessments were done before dosing on day 1 of the IR phase and included: the BPI (12-item instrument to assess pain intensity [from 0 = no pain to 10 = pain as bad as you can imagine], pain relief [0% to 100%], and pain interference with various aspects of the patient's life [from 0 = no interference to 10 = complete interference]) [13], the Mini-Mental State Examination (MMSE; 0–30, higher scores indicate better cognitive performance) [14], and the Eastern Cooperative Oncology Group (ECOG) performance status score (0–4, higher scores indicate poorer performance) [15].

Primary equivalence of efficacy was assessed using the BPI assessment of 'worst pain in the past 24 hours' on an 11-point scale ranging from 0 (no pain) to 10 (worst pain imaginable). This was selected as the primary efficacy measure because evidence suggests that severely intense pain is what interferes most significantly with activities of daily life [13]. Secondary endpoints included other assessments of pain from the BPI (completed by the investigator in consultation with the patient); investigator and patient global assessments of treatment effectiveness (1 = poor, 2 = fair, 3 = good, 4 = very good, 5 = excellent); MMSE; ECOG; time to dose stabilization; and usage and dose of breakthrough pain medication.

Patients rated BPI 'worst pain in the past 24 hours' once daily (just before taking the mid-morning dose of study medication) and 'pain now' twice daily (AM and PM; just before taking the mid-morning and evening doses) throughout the study and recorded responses in daily diaries. The other BPI assessments, plus investigator and patient global evaluations of treatment effectiveness, MMSE, and ECOG, were evaluated at the end of both phases.

Adverse events (AEs) and concomitant medications were recorded throughout the study. Physical examinations were carried out at baseline and at the end of each study phase. Blood and urine samples were collected for laboratory analysis (hematology, serum biochemistry, and urinalysis) at baseline and at the end of the SR phase.

Patients were contacted daily during the IR phase and every 3 to 4 days during the SR phase (preferably by home or clinic visit, or by telephone if appropriate). Contact was also made 3 days after the last dose of study drug to determine the patient's AE status.

### Statistical analysis

Assuming variability (standard deviation, SD) in the primary efficacy variable of 2.0 [16] and a 30% dropout/non-evaluable rate, a sample size of approximately 70 patients per treatment group was required (140 total). This would provide 90% power to detect equivalence with an equivalence limit of 1.5. A planned blinded re-estimation of the optimal sample size was done by an appointed independent person after 55 patients had completed the study, the variability for the primary efficacy measure was estimated using their data, and the sample size was increased to 170 patients. Subsequently, an unplanned, blinded re-estimation of the variability of the primary variable for both phases was done after 120 patients had completed the study, and as a result, the sample size was increased to 200 patients.

All statistical analyses were pre-specified. All efficacy variables were analyzed using the intent-to-treat (ITT) population, which included all patients who took at least 1 dose of study medication and had at least 1 assessment from each study phase. A per-protocol (PP) analysis, excluding data from patients with major protocol deviations, was also done. All patients taking at least 1 dose of study medication were included in the analysis of safety. All efficacy analyses included a pre-specified adjustment for baseline value and country to control for potentially confounding variables.

The primary efficacy analysis used the mean of the last 2 post-baseline recorded values (or the last value if only 1 was available) for BPI 'worst pain in the past 24 hours'. The primary measure was calculated separately for the IR and SR phases (there were therefore two primary outcomes). An analysis of covariance (ANCOVA) was used to calculate the 95% two-sided confidence interval (CI) for the difference between the adjusted means for the 2 treatments at endpoint of each phase. An equivalence test was used to compare the mean scores of the primary efficacy measure at the end of each phase [17-19]. The 2 treatments, within a phase, were considered equivalent if the 95% two-sided CI for the treatment difference was within a -1.5 and 1.5 point difference on the BPI scale. This particular CI range was selected prospectively because it was considered clinically relevant through consultation with physicians who specialize in analgesia.

For secondary endpoints (including BPI 'pain at its least in past 24 hours', 'pain on average pain', 'pain now' (AM and PM), 'pain relief in past 24 hours', 'pain interference in past 24 hours', MMSE, and ECOG) an ANCOVA model was used to calculate 95% two-sided CIs for the difference between the adjusted treatment means at the end of the IR and SR phases. The adjusted means were estimated by the same approach used for the primary endpoint, with the baseline score designated as a covariate; however, no equivalence limits were specified for the secondary endpoints. Time to dose stabilization during both study phases was analyzed by the log-rank test. Summary statistics with 95% CIs were produced for investigator and patient global evaluations of treatment effectiveness and to assess the number of medication doses taken for breakthrough pain in the last 2 days of each phase. *P *values (not adjusted for multiple comparisons) are reported for secondary endpoints. Statistical significance was declared at the α = 0.05 level.

## Results

### Study population

**Figure 2:**
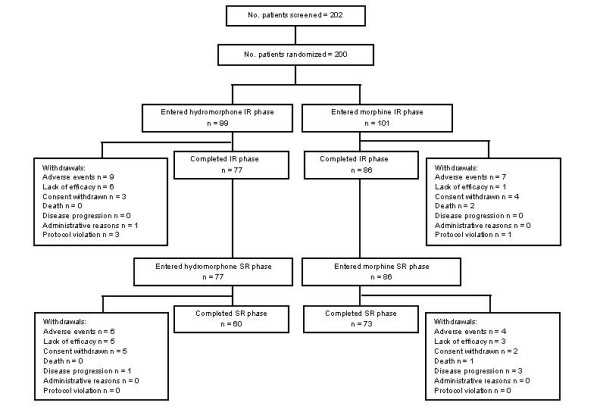
**Patient disposition.** IR, immediate-release; SR, sustained-release.

202 patients were enrolled into the study (Belgium, n = 29; Canada, n = 10; France, n = 7; Germany, n = 15; Netherlands, n = 29; Spain, n = 41; Sweden, n = 11; UK, n = 60). 200 patients were randomized and took study medication. 163 patients (81.5%) completed the IR phase and 133 (66.5%) completed the SR phase. Figure 2 is a flow diagram of patient entry and completion and the reasons for premature discontinuation. Withdrawal from the study owing to lack of efficacy was more common in patients randomized to hydromorphone (n = 11) compared with morphine (n = 4). The mean age of participants overall was 59.8 years. 98.5% of patients were Caucasian and 51% were female. The most common types of cancer were breast (28%), lung (20%), and gastrointestinal (16%). For 133 (67%) patients, the predominant pain location was bone or soft tissue; 34 (17%) others had mixed pain, and 33 (17%) had visceral pain. No patient had neuropathic pain. The most common pain medications used at study entry were morphine (n = 124 [62%]) and tramadol (n = 38 [19%]). The baseline demographics and clinical characteristics were similar for both treatment groups (Table 2).

**Table 2:** Demographic and baseline clinical characteristics

**Characteristic**	**Hydromorphone (N = 99)**	**Morphine (N = 101)**
Mean (SD) age, years	60.7 (12.50)	59.0 (11.36)

Gender, % male	46.5	51.5

Race, % Caucasian/Black/Asian/Other	100/0/0/0	97/0/2/1

Mean (SD) height, cm	166.7 (9.33)	167.3 (10.47)

Mean (SD) weight, kg	66.3 (15.33)	67.4 (13.33)

Mean (SD) BMI, kg/m^2^	23.8 (5.14)	24.2 (5.06)

Cancer type, n (%)		
Breast	23 (23.2)	33 (32.7)
Lung	20 (20.2)	19 (18.8)
Genitourinary	18 (18.2)	12 (11.9)
Gastrointestinal	17 (17.2)	15 (14.9)
Oral cavity	3 (3.0)	3 (3.0)
Lymphoma	3 (3.0)	0
Leukemia	1 (1.0)	2 (2.0)
Bone	1 (1.0)	1 (1.0)
Other	13 (13.1)	16 (15.8)

Predominant pain type, n (%)		
Bone or soft tissue	61 (61.6)	72 (71.3)
Mixed	19 (19.2)	15 (14.9)
Visceral	19 (19.2)	14 (13.9)

Mean (SD) MMSE score*	28.5 (2.3)	28.8 (2.0)

Mean (SD) ECOG score	1.6 (0.8)	1.6 (0.9)

Abbreviations: BMI, body mass index; ECOG, Eastern Cooperative Oncology Group; MMSE, Mini-Mental State Examination; SD, standard deviation

*Hydromorphone, n = 75; morphine, n = 73

### Efficacy

#### BPI 'worst pain in the past 24 hours' (primary endpoint)

**Figure 3:**
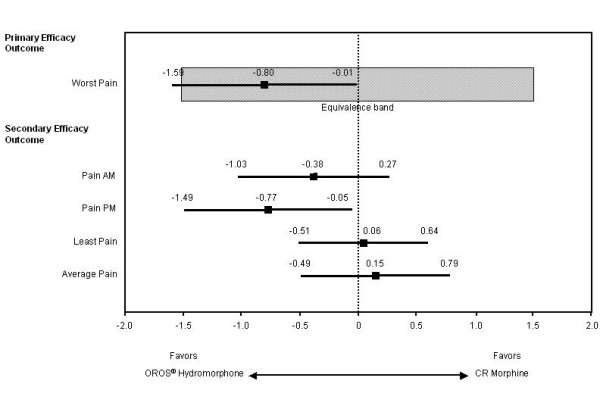
Least-squares mean differences and 95% confidence intervals between the OROS^® ^hydromorphone and controlled-release (CR) morphine groups at end of the sustained-release (SR) phase.

In each study phase, mean values for BPI 'worst pain in the past 24 hours' decreased with both hydromorphone and morphine treatments (Table 3). The least-squares (LS) mean (95% CI) differences between the hydromorphone and morphine groups were 0.2 points (-0.4, 0.9) in the IR phase and -0.8 points (-1.6, -0.01) in the SR phase (ITT population). In the IR phase, the 95% CI was within the -1.5 to 1.5 equivalency range (-0.4 to 0.9), indicating that IR hydromorphone and IR morphine were equivalent in this primary efficacy outcome measure. In the SR phase, the upper limit of the 95% CI was within the 1.5 boundary (-0.01), but the lower limit was less than -1.5 (-1.6), indicating that the SR formulations were not equivalent but that the negative direction of the mean difference was in favor of OROS^® ^hydromorphone (Figure 3). Results of the analysis of the primary efficacy variable were similar when using data from the PP population.

**Table 3:** BPI scores at baseline, end of IR phase, and end of SR phase (ITT population)

**Variable**	Baseline* Mean (SD)	**End of IR phase^† ^LS mean (SE)**	**End of SR phase^‡ ^LS mean (SE)**
Worst pain			
Hydromorphone	6.3 (2.7)	5.0 (0.3)	3.5 (0.3)
Morphine	6.2 (2.5)	4.8 (0.3)	4.3 (0.3)

Least pain			
Hydromorphone	2.7 (2.5)	1.8 (0.2)	1.8 (0.2)
Morphine	2.9 (2.7)	2.2 (0.2)	1.8 (0.2)

Average pain			
Hydromorphone	5.0 (2.1)	3.6 (0.2)	3.4 (0.3)
Morphine	5.1 (2.1)	3.6 (0.2)	3.2 (0.3)

Pain now AM			
Hydromorphone	4.0 (2.7)	3.3 (0.3)	2.4 (0.3)
Morphine	4.1 (2.4)	3.4 (0.3)	2.8 (0.3)

Pain now PM			
Hydromorphone	4.8 (3.0)	3.6 (0.3)	2.6 (0.3)^§^
Morphine	4.2 (2.5)	3.7 (0.3)	3.4 (0.3)

Abbreviations: BPI, Brief Pain Inventory; CR, controlled-release; IR, immediate-release; LS, least-squares; SD, standard deviation; SE, standard error; SR, sustained-release

*Hydromorphone, n = 99; morphine, n = 101

†IR hydromorphone, n = 99; IR morphine, n = 101

‡OROS^® ^hydromorphone, n = 77; CR morphine, n = 86

§p = 0.0372 versus CR morphine

#### Secondary endpoints

**Figure 4:**
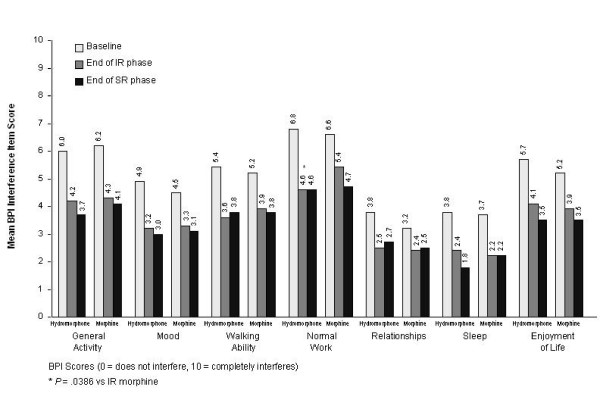
**Mean Brief Pain Inventory (BPI) interference scores at baseline, end of immediate-release (IR) phase, and end of sustained-release (SR) phase.** Values at baseline represent means; values at other time points represent least-squares means. At baseline: hydromorphone, n = 99; morphine, n = 101. In IR phase: IR hydromorphone, n = 99; IR morphine, n = 101. In SR phase: OROS^® ^hydromorphone, n = 77; controlled-release (CR) morphine, n = 86.

At the end of the IR phase, mean scores for the secondary efficacy variables (other assessments of pain from the BPI [Table 3 and Figure 4], and MMSE and ECOG scores [data not shown]) were similar for IR hydromorphone and IR morphine, except for BPI 'pain interference with normal work', which favored IR hydromorphone (Figure 4; *p *= 0.0386).

**Figure 5:**
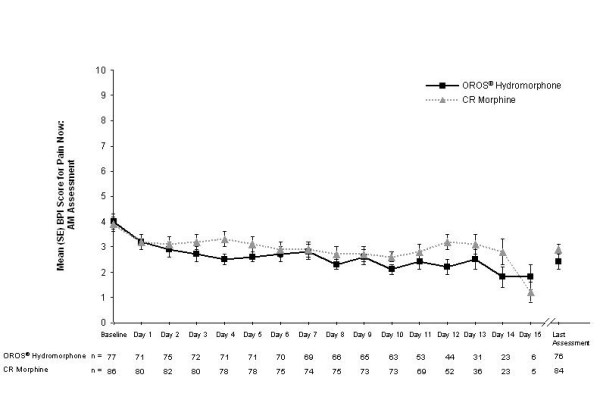
**Mean (standard error, SE) pain now scores in the morning during the sustained-release (SR) phase.** Pain was rated using the 11-point Brief Pain Inventory (BPI), ranging from 0 (no pain) to 10 (worst pain imaginable). CR, controlled-release.

**Figure 6:**
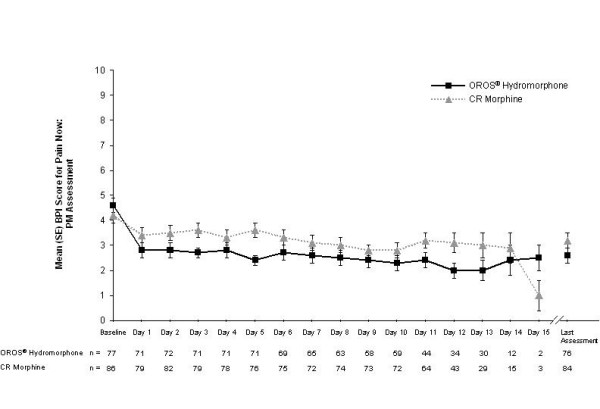
**Mean (standard error, SE) pain now scores in the evening during the sustained-release (SR) phase.** Pain was rated using the 11-point Brief Pain Inventory (BPI), ranging from 0 (no pain) to 10 (worst pain imaginable). CR, controlled-release.

At the end of the SR phase, scores were similar between treatment groups for BPI items 'least pain', 'average pain', and 'pain now AM'; however, 'pain now PM' was significantly lower in the OROS^® ^hydromorphone group compared with the CR morphine group (LS mean [SE] scores, 2.6 [0.3] versus 3.4 [0.3], respectively; *p *= 0.0372) (Table 3). LS mean differences (95% CI) between treatment groups were 0.06 (-0.51, 0.64), 0.15 (-0.49, 0.79), -0.38 (-1.03, 0.27), and -0.77 (-1.49, -0.05) for least, average, morning, and evening pain, respectively (Figure 3). Figures 5 and 6 show the consistency of response to both OROS^® ^hydromorphone and morphine throughout the SR phase, during both the morning and evening assessments of BPI 'pain now' scores.

LS mean BPI 'pain relief' at endpoint was 69.7% for the OROS^® ^hydromorphone group and 70.0% for the CR morphine group (95% CI for treatment difference, -8.11, 7.54). BPI 'pain interference' scores were also similar between treatment groups at endpoint, (Figure 4), as were LS mean ECOG performance status scores (1.8 versus 1.7 in the OROS^® ^hydromorphone and CR morphine groups, respectively; 95% CI for treatment difference, -0.13, 0.26), and LS mean MMSE scores (28.9 versus 29.2; 95% CI for treatment difference, -1.19, 0.51).

**Figure 7:**
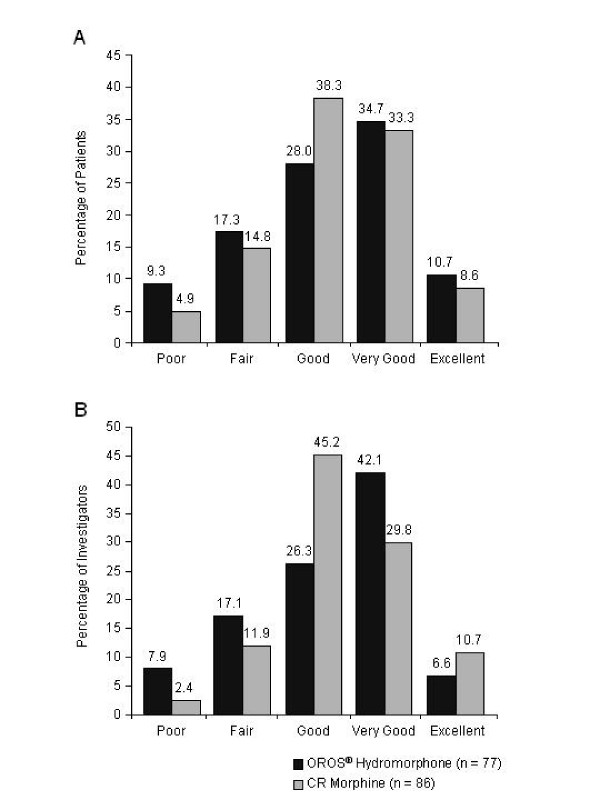
Patient (A) and investigator (B) global evaluations of treatment effectiveness at the end of the sustained-release (SR) phase. CR, controlled-release.

The majority of both investigators and patients (> 70%) rated the treatments as good, very good, or excellent at the end of each study phase (Figure 7). Mean ± SD patient global assessment scores at the end of the SR phase were 3.2 ± 1.14 for the OROS^® ^hydromorphone group and 3.3 ± 0.98 for the CR morphine group (*p *= 0.6696); mean ± SD investigator global assessment scores were 3.2 ± 1.07 and 3.3 ± 0.91, respectively (*p *= 0.4760).

In both treatment phases, patients in the hydromorphone group took longer than those in the morphine group to achieve stable doses (*p *< 0.001). The number of patients requiring dose-level changes during the SR phase was similar for both groups (19 of 77 [24.7%] OROS^® ^hydromorphone recipients and 23 of 86 [26.7%] CR morphine recipients). Comparison of dosage levels at the end of the SR phase generally showed that similar proportions of patients received OROS^® ^hydromorphone or CR morphine at each of the 5 (approximate 1:5 hydromorphone to morphine) lower dosage levels, although more patients in the OROS^® ^hydromorphone group (10% versus 2% in the CR morphine group) received the highest dosage level evaluated in this study (Table 4).

**Table 4:** Dose levels at end of SR phase

	**OROS^® ^hydromorphone (N = 77)**		**CR morphine (N = 86)**	
**Dose level**	**Dose (mg) every 24 hours**	**Number of subjects (%)**	**Dose (mg) every 12 hours**	**Number of subjects (%)**

1	16	22 (28.6)	30	25 (29.1)

2	24	14 (18.2)	60	23 (26.7)

3	32	18 (23.4)	90	15 (17.4)

4	48	10 (13.0)	120	13 (15.1)

5	72	5 (6.5)	175	8 (9.3)

6	96	8 (10.4)	260	2 (2.3)

Abbreviations: CR, controlled-release; SR, sustained-release

In the IR phase, 61.6% of patients taking IR hydromorphone and 52.5% of those receiving IR morphine required medication for breakthrough pain on the last 2 days of the phase. In the SR phase, the corresponding percentages in the OROS^® ^hydromorphone and CR morphine groups were 48.1% and 48.8%, respectively. The mean (SD) numbers of breakthrough pain medication doses taken on the last 2 days were as follows: IR phase, 1.8 (2.2) and 1.3 (1.8) for the IR hydromorphone and IR morphine groups, respectively; SR phase, 1.6 (2.2) and 1.4 (1.9) for the OROS^® ^hydromorphone and CR morphine groups, respectively. The mean total doses of breakthrough pain medication as a percentage of the mean daily consumption were 39.4% and 27.1% for hydromorphone and morphine in the IR phase, and 13.8% and 10.3% for OROS^® ^hydromorphone and CR morphine in the SR phase. There were no statistically significant treatment differences in the numbers of patients who took breakthrough pain medication or in the amount of medication taken in either phase.

### Safety

The overall safety profiles of hydromorphone and morphine were comparable, with a similar number of patients in each group reporting AEs. Overall, irrespective of study phase, 81 of 99 (81.8%) hydromorphone-treated patients and 90 of 101 (89.1%) morphine-treated patients reported at least 1 AE.

The most commonly reported AEs during the IR and SR phases of the study are listed in Table 5. The types of AEs were typical of those generally associated with opioid use. The incidence of constipation was higher with hydromorphone than morphine in both phases of the study (23.2% versus 10.9% in the IR phase and 39.0% versus 22.1% in the SR phase). In the SR phase, the incidences of vomiting, nausea, and somnolence were higher with CR morphine than with OROS^® ^hydromorphone (22.1% versus 9.1%, 29.1% versus 19.5%, and 14.0% versus 10.4%, respectively). Most AEs were mild or moderate in severity and no clear treatment difference was seen in the relative severity of the most commonly reported AEs. Approximately 30% of patients with AEs reported events that were considered unlikely to be related or not related to study therapy.

**Table 5:** Adverse events reported by at least 5% of patients in any group during the IR or SR phase

	**IR phase**		**SR phase**	
**Adverse event**	**Hydromorphone (N = 99)**	**Morphine (N = 101)**	**OROS^® ^hydromorphone (N = 77)**	**CR morphine (N = 86)**

Constipation	23 (23.2%)	11 (10.9%)	30 (39.0%)	19 (22.1%)

Nausea	18 (18.2%)	23 (22.8%)	15 (19.5%)	25 (29.1%)

Vomiting	16 (16.2%)	19 (18.8%)	7 (9.1%)	19 (22.1%)

Somnolence	11 (11.1%)	11 (10.9%)	8 (10.4%)	12 (14.0%)

Dizziness	8 (8.1%)	6 (5.9%)	4 (5.2%)	8 (9.3%)

Headache	8 (8.1%)	6 (5.9%)	3 (3.9%)	2 (2.3%)

Diarrhea	8 (8.1%)	1 (1.0%)	7 (9.1%)	2 (2.3%)

Pruritus	4 (4.0%)	5 (5.0%)	3 (3.9%)	5 (5.8%)

Asthenia	5 (5.1%)	1 (1.0%)	6 (7.8%)	4 (4.7%)

Fatigue	3 (3.0%)	3 (3.0%)	4 (5.2%)	6 (7.0%)

Confusion	2 (2.0%)	2 (2.0%)	7 (9.1%)	2 (2.3%)

Anemia	2 (2.0%)	1 (1.0%)	3 (3.9%)	6 (7.0%)

Anorexia	1 (1.0%)	4 (4.0%)	2 (2.6%)	5 (5.8%)

Insomnia	1 (1.0%)	2 (2.0%)	5 (6.5%)	4 (4.7%)

Peripheral edema	0	3 (3.0%)	1 (1.3%)	8 (9.3%)

Pyrexia	2 (2.0%)	1 (1.0%)	4 (5.2%)	2 (2.3%)

Anxiety	1 (1.0%)	1 (1.0%)	5 (6.5%)	1 (1.2%)

Abbreviations: CR, controlled-release; IR, immediate-release; SR, sustained-release

Serious AEs were reported for 24 patients during the double-blind period (by 5 patients in each treatment group in the IR phase, and by 7 patients in the OROS^® ^hydromorphone group and 9 in the CR morphine group in the SR phase [2 patients had serious AEs in both phases]). Many of the serious AEs were associated with the underlying disease, although approximately one third were considered definitely or probably related to study therapy. Three patients in the morphine group died during the study (2 during the IR phase, 1 during the SR phase). None of the deaths was considered related to the study medication.

Twenty-six patients withdrew prematurely from the study because of AEs, 16 during the IR phase (9 hydromorphone, 7 morphine) and 10 during the SR phase (6 OROS^® ^hydromorphone, 4 CR morphine). There was no statistically significant difference between treatment groups in the time to withdrawal, irrespective of phase (IR phase, *p *= 0.6537; SR phase, *p *= 0.2827). The use of concomitant medications, including the use of non-opioid analgesics, was similar for both treatment groups in both the IR and SR phases.

## Discussion

This was a short-term, randomized, double-blind comparative study testing the clinical equivalence of IR and SR formulations of hydromorphone and morphine in patients with chronic cancer pain. In each study phase (IR and SR), mean values for BPI 'worst pain in the past 24 hours' (the primary efficacy endpoint) decreased with both hydromorphone and morphine. In the IR phase, the LS mean difference between the treatment groups was 0.2 points, and the 95% CI (-0.4, 0.9) was within the pre-specified equivalency range of -1.5 to 1.5. In the SR phase, the LS mean difference was -0.8 points in favor of OROS^® ^hydromorphone. The upper limit of the 95% CI (-0.01) was within the 1.5 boundary, but the lower limit (-1.6) was less than the -1.5 limit. Therefore, according to the pre-specified criteria, equivalence was not demonstrated; the direction of the difference was in favor of OROS^® ^hydromorphone. The BPI item 'worst pain in the past 24 hours' was selected as the primary endpoint of the trial based on previous studies showing it to be the most sensitive BPI measure in the clinical trial setting [13,20-22].

The results of the present study further suggest that OROS^® ^hydromorphone provides consistent pain relief over 24 hours, and that pain levels in the evening were significantly lower after OROS^® ^hydromorphone compared with CR morphine treatment. This finding reflects the 'pain now PM' measure being scored at the end of morphine dosing (i.e. trough levels) but at the midpoint of hydromorphone dosing. The comparable results between groups for the other secondary efficacy variables (including assessments of pain interference with physical activity and social function, performance status, and cognition) support similarity in the treatment efficacy. Withdrawal from the study owing to lack of efficacy was more common in patients randomized to hydromorphone (n = 11) compared with morphine (n = 4). This is a potentially negative finding for hydromorphone; however, the numbers are too low to draw any definite conclusions.

Results of the dose analysis were generally consistent with those of previous reports, which indicate that most cancer pain can be controlled with ≤ 240 mg of oral morphine daily [23]. In the present study, 83% of the OROS^® ^hydromorphone group and 88% of the CR morphine group received the equivalent of morphine 240 mg/day or less.

The frequency of breakthrough medication use remained stable throughout both phases in the morphine group (52.5% in the IR phase, 48.8% in the SR phase), but decreased from the IR phase to the SR phase in the OROS^® ^hydromorphone group (from 61.6% to 48.1%). At the end of the SR phase, there was no significant difference between the groups in use of breakthrough medication.

The occurrence of 3 deaths and 23 serious AEs during the study was not unexpected, given the severity of the patients' conditions and the progressive nature of the disease. In fact, many of the serious AEs were associated with the underlying disease.

The participants in this study are representative of the overall population afflicted with chronic cancer pain. The mean baseline BPI score for 'worst pain in the past 24 hours' was 6.3 on an 11-point scale, a score indicative of moderate to severe pain [16]. This is comparable to baseline values in other studies of cancer pain, for which the mean score was 6.99 [24]. Baseline cognition was similar to that of healthy controls (mean MMSE score, 28.3) [25], and the lack of change observed in both treatment groups probably reflects the fact that the MMSE was designed to measure cognition in Alzheimer's disease [14] and was not intended to measure cognition in non-demented subjects.

The major positive aspect of this study was the robust design: it was multicenter, randomized, double-blind, and active controlled. It also used a widely utilized and well validated primary outcome measure, the BPI. On the negative side, there was a relatively poor completion rate, with only 60 out of 99 patients (60.6%) randomized to hydromorphone and 73 out of 101 patients (72.3%) randomized to morphine completing both treatment phases. However, this completion rate was reasonably good when taking into consideration that this study was carried out in patients with cancer which was advanced enough to require strong opioid analgesics. In addition, a dropout rate of at least 30% was anticipated. Furthermore, the duration of the study was relatively short (up to 24 days), so it was not possible to make evaluations of the sustained efficacy of the formulations.

Two previous randomized, double-blind, crossover studies have compared CR formulations of hydromorphone and morphine in patients with cancer pain [26,27]. Moriarty et al (N = 100) found no significant differences between treatment groups for any outcome measure, and AEs were mild and infrequent [27]. In the other study (N = 87), patients treated with hydromorphone had significantly higher pain scores and required more doses of rescue analgesia than patients receiving morphine [26]. In addition, more patients in the hydromorphone group withdrew owing to AEs or inadequate analgesia (n = 16 versus n = 2 on morphine). Although it is difficult to directly compare the results of these studies with those of the current study, the observed differences may reflect, at least in part, the more consistent delivery of hydromorphone with the OROS^® ^formulation relative to the dosing used in those studies. The consistent release of hydromorphone over 24 hours has been demonstrated in previous studies, in which steady-state plasma concentrations were achieved by 48 hours and sustained throughout the dosing interval [8,28].

Practicing clinicians are acutely aware of the need for multiple pain management alternatives, because it is clear that the response to a particular opioid agent varies among individuals. Although our understanding of the causes of individual differences remains incomplete, receptor and receptor-binding studies suggest that they may result not only from differences in the class of opioid receptors to which each agent binds (morphine binds to mu [primary] and kappa [lesser extent] [29]; hydromorphone binds to mu [primary] and delta [lesser extent] [30]), but also from inter-individual differences in opioid receptors (several receptor subtypes have been identified [mu 1, mu 2, mu 3, delta 1, delta 2, kappa 1, kappa 2, kappa 3]) and the location of these variants within the nervous system [31-33]. Therefore, clinicians may need to utilize different opioids to achieve maximum efficacy for each patient. In addition, some studies have supported the use of opioid rotation, switching from one opioid to another when treatment-limiting toxicity results in reduced responsiveness [34-37]. Based on numerous observations that individual response varies greatly from opioid to opioid, changing to a different drug may provide an improved balance between analgesia and adverse effects; as a result, there is a need for additional long-acting opioids on the market.

Long-acting opioid formulations such OROS^® ^hydromorphone have the potential to improve the overall management of chronic pain. Such formulations provide more consistent opioid plasma concentrations, avoiding the peaks and troughs associated with short-acting agents, and result in well-tolerated, around-the-clock pain control with fewer daily doses [38,39].

## Conclusion

This study demonstrates that while IR hydromorphone is equivalent to IR morphine for relieving chronic cancer pain, equivalence was not demonstrated for OROS^® ^hydromorphone and CR morphine in the SR phase of the study. The direction of the mean difference between the treatments, and the out-of-range lower limit of the 95% CI was in favor of OROS^® ^hydromorphone on the primary endpoint 'worst pain'. 'Pain now PM' scores were also significantly lower for those taking OROS^® ^hydromorphone at the end of the SR phase (although the timing of this measure coincided with a trough in the dosing levels for the twice-daily morphine compared with the once-daily OROS^® ^hydromorphone), and BPI 'interference with normal work' scores were significantly lower for IR hydromorphone. None of the other secondary efficacy analyses showed significant treatment differences in either phase. The overall safety profiles of the 2 treatments were generally similar and most AEs were typical of opioid analgesic therapy.

## Competing interests

MH was a paid advisor for Janssen-Cilag.

## Authors' contributions

MH was an investigator in this study and was involved in revising this manuscript for important intellectual content. JT contributed to the analysis of the study and reviewed the manuscript.

## Pre-publication history

The pre-publication history for this paper can be accessed here:


